# Synergic silencing of costimulatory molecules prevents cardiac allograft rejection

**DOI:** 10.1186/1479-5876-12-142

**Published:** 2014-05-22

**Authors:** Xusheng Zhang, Yanling Liu, Guangfeng Zhang, Jun Shi, Xiao Zhang, Xiufen Zheng, Alex T Jiang, Zhu-Xu Zhang, Nathan Johnston, King Sun Siu, Ruiqi Chen, Dameng Lian, David Koos, Douglas Quan, Wei-Ping Min

**Affiliations:** 1 Department of Surgery, Pathology, and Ocology, University of Western Ontario, London, Canada; 2 Multi-Organ Transplant Program, London Health Sciences Centre, London, Canada; 3 Jiangxi Academy of Medical Sciences, The First Affiliated Hospital, and Institute of Immunotherapy of Nanchang University, Nanchang, China; 4 Department of Rheumatology, Guangdong Academy of Medical Sciences, Guangdong General Hospital, Guangdong, China; 5 Regen BioPharma, San Diego, USA

**Keywords:** Co-stimulatory molecule, Heart transplantation, RNA interference, Tolerance

## Abstract

**Background:**

While substantial progress has been made in blocking acute transplant rejection with the advent of immune suppressive drugs, chronic rejection, mediated primarily by recipient antigen presentation, remains a formidable problem in clinical transplantation. We hypothesized that blocking co-stimulatory pathways in the recipient by induction of RNA interference using small interference RNA (siRNA) expression vectors can prolong allogeneic heart graft survival.

**Method:**

Vectors expressing siRNA specifically targeting CD40 and CD80 were prepared. Recipients (BALB/c mice) were treated with CD40 and/or CD80 siRNA expression vectors via hydrodynamic injection. Control groups were injected with a scrambled siRNA vector and sham treatment (PBS). After treatment, a fully MHC-mismatched (BALB/c to C57/BL6) heart transplantation was performed.

**Result:**

Allogeneic heart graft survival (>100 days) was approximately 70% in the mice treated simultaneously with CD40 and CD80 siRNA expression vectors with overall reduction in lymphocyte interstitium infiltration, vascular obstruction, and edema. Hearts transplanted into CD40 or CD80 siRNA vector-treated recipients had an increased graft survival time compared to negative control groups, but did not survive longer than 40 days. In contrast, allogenic hearts transplanted into recipients treated with scrambled siRNA vector and PBS stopped beating within 10–16 days. Real-time PCR (RT-PCR) and flow cytometric analysis showed an upregulation of FoxP3 expression in spleen lymphocytes and a concurrent downregulation of CD40 and CD80 expression in splenic dendritic cells of siRNA-treated mice. Functional suppressive activity of splenic dendritic cells (DCs) isolated from tolerant recipients was demonstrated in a mixed lymphocyte reaction (MLR). Furthermore, DCs isolated from CD40- and CD80-treated recipients promoted CD4 + CD25 + FoxP3+ regulatory T cell differentiation in vitro.

**Conclusion:**

This study demonstrates that the simultaneous silencing of CD40 and CD80 genes has synergistic effects in preventing allograft rejection, and may therefore have therapeutic potential in clinical transplantation.

## Introduction

Dendritic Cells (DCs) are the most potent antigen-presenting cells (APCs), having a role on both priming the adaptive immune response and induction of immunological tolerance [1-3]. DCs can be either immunostimulatory or immunoregulatory; it has been demonstrated that the properties of DCs depend on maturation status, phenotype and source of origin. In general, mature DCs express high levels of CD11C, major histocompatibility complex class II (MHC II) and the costimulatory molecules CD40 and CD80. DCs that inhibit immune responses have been described as immature, having plasmacytoid morphology, or being alternatively activated. Collectively, suppressive DCs have been termed “tolerogenic DCs”. Previous studies have demonstrated that donor-specific, allogeneic tolerogenic DCs can enhance survival of transplanted grafts [4,5].

T cells require two signals to become fully activated. The first signal, which is antigen-specific, is provided through the T cell receptor which interacts with peptide-MHC molecules on the membrane of APCs. A second signal, the co-stimulatory signal, is antigen nonspecific and is provided by the interaction between co-stimulatory molecules expressed on the membrane of APCs and the T cells. T cell co-stimulation is necessary for T cell proliferation, differentiation and survival. Activation of T cells without co-stimulation may lead to T cell anergy, T cell deletion or the development of immune tolerance [3,6,7].

Multiple costimulatory pathways are involved in primary T cells activation. CD28/Cytotoxic T-Lymphocyte Antigen 4 (CTLA4) binding to CD80/CD86 was the first costimulatory pathway identified and is one of the most potent and best characterized of costimulatory interactions [8,9]. CD80/CD86 on APCs ligated with their receptors CD28/CTLA4 on T cells could regulate T cell responses. Interaction through CD80/CD86-CD28 pathway is crucial for enhancing T cells activation and survival, however, the CD80/CD86-CTLA4 pathway is mainly for regulating inhibitory T cell responses. CD40 is a type I transmembrane protein which belongs to the TNF receptor superfamily and is found to be expressed on all types of antigen-presenting cells (APCs), particularly on DCs [10]. CD40 on DCs bind to T cell CD40 ligand (CD40L) and activate T cells by upregulating CD80 and CD86 on DCs. As well, this interaction can induce high levels of the proinflammatory cytokine IL-12, which is critical for the development of Th1 type immune responses [11,12]. The blockade of the CD40-CD40L pathway will result in a deficiency in APC interaction, which will also lead to the global failure of T cell activation [13,14]. Costimulation blockade targeting either CD28-CD80/CD86 or CD40-CD40L alone rarely gave durable allograft survival. Therefore, simultaneous blockade of these two pathways has synergistic function in promoting allograft tolerance [15].

Gene silencing by using small interfering RNA (siRNA) is capable of specifically blocking gene expression in mammalian cells without triggering the nonspecific panic response [16,17]. The strategies of using siRNA have been successful in inducing therapeutic benefits in animal models of various diseases and are currently in clinical trials [18-23]. To date, blockade of the costimulatory molecules is being aggressively pursued as a tolerance-inducing strategy [24]. Inhibition of this bidirectional interaction not only suppresses T cell responses [25] and Th2 cytokines, but also actively generates regulatory T (Treg) cells [26]. In the present study, we investigated the feasibility of silencing both CD40 and CD80 expression by siRNA treatment in the recipient to induce longer cardiac allograft survival.

## Methods and material

### Mice

Male 8–10 week old C57BL/6 and BALB/c mice (Charles River Canada, Saint-Constant, Canada) were used as donors and recipients, respectively. Animals were housed under conventional conditions at the Animal Care Facility, University of Western Ontario, and were cared for in accordance with the guidelines established by the Canadian Council on Animal Care.

### DCs culture

DCs were cultured from bone marrow progenitor cells as previously described [27]. Briefly, bone marrow cells were flushed from the femurs and tibias of C57BL/6 mice then washed and cultured in 6-well plates supplemented with 10 ng/ml of recombinant GM-CSF and recombinant mouse IL-4 (Peprotech, Rocky Hill, NJ, USA). All cultures were incubated at 37°C in 5% humidified CO_2_.

### CD40 and CD80 siRNA and expressed siRNA vector constructs

For *in vitro* studies, CD40 and CD80 siRNA were synthesized by Dharmacon (Chicago, IL). The sequence of CD40 siRNA used was UUCUCAGCCCAGUGGAACA, and the sequence of CD80 used was GUGUGGCCCGAGUAUAAGA. The DCs were transfected with siRNA by using lipofectamine 2000 (Life technologies, Burlington).

For *in vivo* studies, the siRNA expression vector was constructed as previously described [28,29]. The oligonucleotides containing target-specific sense and anti-sense sequences of CD40 and CD80 mRNAs were synthesized, annealed and inserted into the pRNAT U6.1 siRNA expression vector utilizing restriction enzyme sites at the end of the strands (Genscript, Piscataway, NJ) to express the siRNAs.

### Heterotopic cardiac transplantation and treatment

Recipients (BALB/c) were treated with CD40 and CD80 siRNA vectors 3 days prior to heart transplantation and 7, 14 and 21 days after transplantation by hydrodynamic injection. 50 μg of CD40 and CD80 siRNA vectors were diluted in 1.6 ml of PBS and rapidly injected into the mice through the tail vein within 5-7s [23,30,31]. A low dose (2Gy) of whole body irradiation was administered to the recipient mice before heart transplantation. Recipient BALB/c (H-2^d^) mice were subjected to intra-abdominal allogeneic cardiac transplantation using the hearts from fully MHC-mismatched C57BL/6 (H-2^b^) mice. Pulsation of cardiac grafts was monitored daily by direct abdominal palpation in a double-blind manner to determine survival/rejection of the cardiac graft.

### Quantitative real-time PCR (RT-PCR)

Total RNA was extracted from cells using Trizol (Invitrogen). Total RNA (3 μg) was used for cDNA synthesize using oligo-(dT) primer and reverse transcriptase (Invitrogen). Primers used to amplify murine CD40, CD80, FoxP3 and GAPDH genes were: CD40, 5′- AGCGGTCCATCTAGGGCAGTGTG -3′ (forward) and 5′- TGGGTGGCATTGG GTCTTCTCA-3′ (reverse); CD80, 5′- GCCTCGCTTCTCTTGGTTG - 3′ (forward), 5′- TTACTGCGCCGAATCCTG-3′ (reverse); FoxP3, 5′- CAGCTGCCTACAGTGCCCCT AG-3′(forward), 5′- CATTTGCCAGCAGTGGGTAG-3′ (Reverse); GAPDH, 5′- TGA TGACATCAAGAAGGTGGTGAA-3′ (forward) and 5′- TCCTTGGAGGCCATGTAG GCCAT -3′ (reverse). Real-time PCR reactions were performed in a Stratagene Mx3000P QPCR System (Agilent Technologies, Lexington, MA) using SYBR green PCR Master Mix (Life technologies) according to manufacture’s protocol. The PCR reaction condition was 95°C for 10 min, and 95°C for 30 sec, 58°C for 45 sec and 72°C for 30 sec (40 cycles).

### Flow cytometry

Characterization of DCs or T cells was performed by flow cytometer (Becton Dickinson, San Jose, CA). All antibodies were purchased from eBioscience, San Diego, CA, unless otherwise indicated.

DCs were stained with FITC- or PE-CD40 and PE-CD80 monoclonal antibodies. For T cells, PE-Cy5-CD4, FITC-FoxP3, and PE-CD25 conjugated anti-mouse monoclonal antibodies were used for staining. Foxp3 expression was assessed by intracellular staining, using Foxp3 Staining Kits (eBioscience). All flow cytometric analysis was performed using appropriate isotype controls (Cedarlane Laboratories).

### Mixed lymphocyte reaction (MLR)

For *in vitro* MLR, T cells (2 × 10^5^/well) from naïve BALB/c mice were plated with DCs cultured from C57BL/6 mice in varying ratios of DC:T cells. For *in vivo* MLR, splenic DCs isolated from tolerant or rejecting recipients (BALB/c) using CD11c MACS beads (Miltenyi Biotec) were irradiated at 30 Gy. T cells (2 × 10^5^/well) from C57BL/6 mice were added to the DC cultures, with the final MLR taking place in 200 μl of complete RPMI 1640 medium (Life Technologies). Cells were cultured at 37°C in a humidified atmosphere of 5% CO2 for 3 days, and pulsed with 1 μCi of [^3^H] thymidine (PerkinElmer, Woodbridge, ON) for the last 18 h of culture. Cells were harvested onto glass fiber filters, and the incorporated radioactivity was quantified using a Wallac Betaplate liquid scintillation counter. Results were expressed as the mean counts per minute (cpm) of triplicate cultures ± SEM.

To determine the ability of Treg to perform the inhibitory MLR splenic T cells (2 × 10^5^/well) from naïve BALB/C mice were used as responder cells. Cultured bone marrow DCs (1 × 10^5^) from naïve C57BL/6 and C3H (third party) mice were used as stimulators. CD4^+^CD25^+^ cells isolated from spleens of tolerant recipient mice using a Treg cells isolation kit (Miltenyi Biotec) were added to the cultures; ratios of Treg:stimulator were 1:100, 1:20, 1:10. Experimental procedures used to incubate and harvest cells were the same as described above.

### Graft histology

At the experimental endpoint, cardiac tissue samples were collected and fixed in 10% buffered formaldehyde and processed for histology examination using standard techniques. Specimens were embedded in paraffin, and sectioned for H&E staining. The microscopic sections were examined in a blinded fashion by pathologist for rejection. Criteria for allograft rejection included the presence of myocardial infarction, lymphocytic infiltration, thrombosis and hemorrhage.

### Statistical analysis

In this study, data were reported as the mean ± SEM. Allograft survival among experimental groups was compared using the log-rank test. Quantitative real-time PCR data were analyzed using one-way ANOVA. Differences with P values less than 0.05 were considered significant.

## Results

### CD40 and CD80 siRNA gene silencing validation in vitro

**Figure 1:**
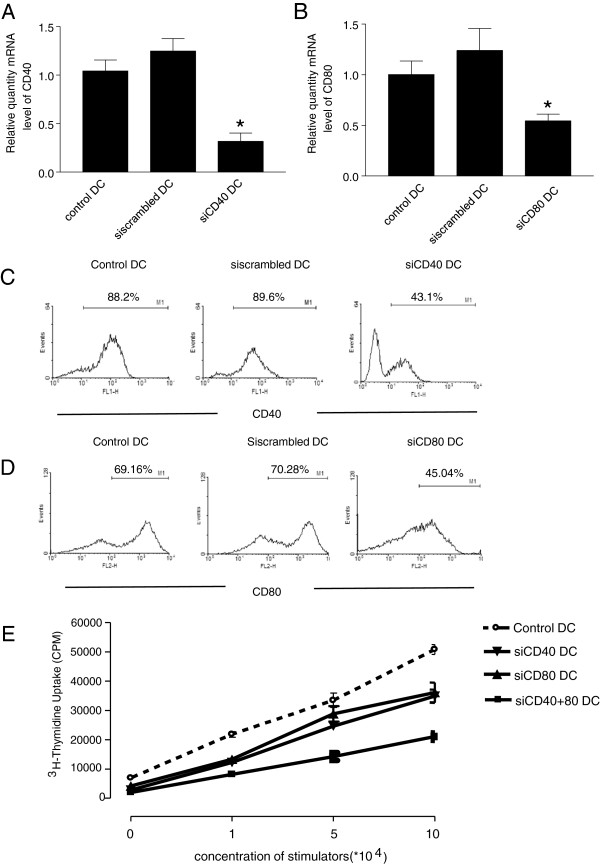
**CD40 and CD80 gene silencing *in vitro *. (A & B)***In vitro* gene silencing determined by quantitative RT-PCR. C57BL/6 mice bone marrow DCs were cultured for 6 days and were transfected with CD40, CD80 or scrambled siRNA using lipofectamine 2000. Non-transfected cells served as a negative control. Twenty-four hours after transfection, LPS was added for another 24h. Forty-eight hours after transfection, cells were harvested and total RNA was extracted. Transcripts of CD40 **(A)** and CD80 **(B)** were determined using quantitative RT-PCR. (*p < 0.01, CD40 or CD80 siRNA vs untransfected or scrambled siRNA transfected cells). **(C & D)***In vitro* gene silencing of CD40 and CD80 detected by flow cytometry DCs were culture and transfected with siRNA as described in **A** &**B**. DCs were harvested and stained with FITC-labeled CD40 and PE-labeled CD80 antibodies. The expression of CD40 **(C)** and CD80 **(D)** was detected by flow cytometry. **(E)** CD40 and CD80 silenced DCs attenuate allogeneic T cell proliferation. Bone marrow DCs were cultured and transfected with CD40 and CD80 siRNA as described in **A** &**B**. Forty-eight hours after transfection, DCs were collected and co-cultured with allogeneic T cells in a 96 well plate at various ratios as indicated. [^3^H] was added 48h after co-culture, and its incorporation was measured as an indicator of T cell proliferation. (*p < 0.01 vs control group).

Mature, immunologically competent DCs are the most efficient APCs. Upon stimulation with antigen, DCs change from immature antigen capturing cells to mature antigen presenting cells and active T cells [32]. Costimulatory molecules CD40 and CD80 are highly expressed on mature DCs. Thus we first validated the efficacy of gene silencing using siRNA specifically targeting the CD40 and CD80 genes in cultured LPS-stimulated mature DCs. We confirmed that both CD40 and CD80 were expressed in the DCs cultured from C57BL/6 bone marrow by quantitative real-time PCR (Figure 1A, B). Forty eight hours after transfecting DCs with CD40 and CD80 siRNAs, CD40 and CD80 gene expression was reduced by approximately 75% and 55%, respectively, when compared with the DCs transfected with scrambled siRNA or untransfected control DCs (Figure 1A-B).

We further confirmed the gene silencing efficiency by flow cytometry. Upon activation by LPS, untreated control DCs and scrambled siRNA transfected DCs highly expressed CD40 (89%) and CD80 (70%), suggesting that these DCs were mature (Figure 1C, D). DCs transfected with CD40 or CD80 siRNA showed decreased CD40 (43%) or CD80 (41%) costimulatory molecule expression.

To evaluate the capacity of DCs to stimulate allogeneic T cells responses after gene silencing of CD40 and CD80, we performed mixed leukocyte reaction (MLR). DCs cultured from C57BL/6 mice transfected with CD40 or CD80 siRNA alone or in combination were used as stimulators, while DCs transfected with scrambled siRNA were used as controls. These DCs were plated and cultured with allogeneic T cells from BALB/c mice (Figure 1E). The results showed that, control DCs initiated a strong allogeneic T cells responses, CD40 or CD80 alone-silenced DCs showed reduced levels of allogeneic T cell responsed, although the differences between the groups did not reach statistical significance. However, silencing both CD40 and CD80 using siRNA significantly inhibited allogeneic T cell proliferation. These data suggested that CD40 and CD80-silenced DCs are immunosuppressive or tolerogenic DCs and fail to stimulate T cell responses.

### CD40 and CD80 gene silencing in vivo

**Figure 2:**
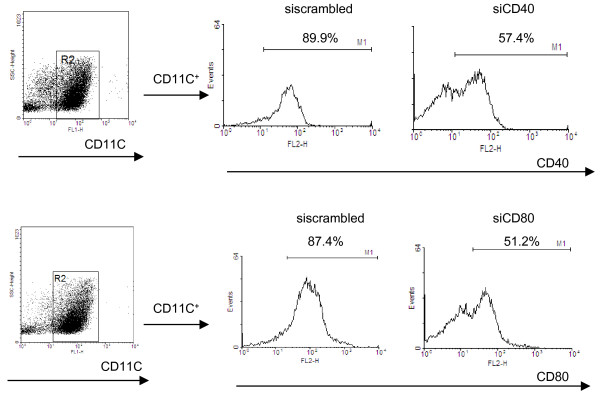
**CD40 and CD80 gene silencing *in vivo*.** Fifty micrograms of CD40 and CD80 siRNA vector or scrambled siRNA control vector were administrated to mice by iv injection. Forty-eight hours after injection, 0.5 mg LPS was administrated by ip injection. Splenic DCs were isolated 24h after LPS injection using MACS beads. Scrambled siRNA vector treated mice served as a control. The expression of CD40 and CD80 in splenic DCs was detected by flow cytometry.

To validate CD40 and CD80 gene silencing efficiency *in vivo*, we treated mice with CD40 or CD80 siRNA vectors using the method of hydrodynamic injection through the tail vein. In order to stimulate spleen DC maturation, the mice were also subsequently treated with LPS. We isolated splenic DCs and performed flow cytometry to detect CD40 and CD80 expression. Mice that were administered scrambled siRNA vector plus LPS showed upregulated expression of CD40 (89%) and CD80 (87%) (Figure 2). Treatment with CD40 or CD80 siRNA vectors significantly decreased CD40 (57%) and CD80 (51%) gene expression. These data demonstrate that the CD40 or CD80 siRNA vectors were capable of knocking down CD40 or CD80 gene expression *in vivo*.

### Prevent cardiac allograft rejection by using CD40 and CD80 siRNA expression vector

**Figure 3:**
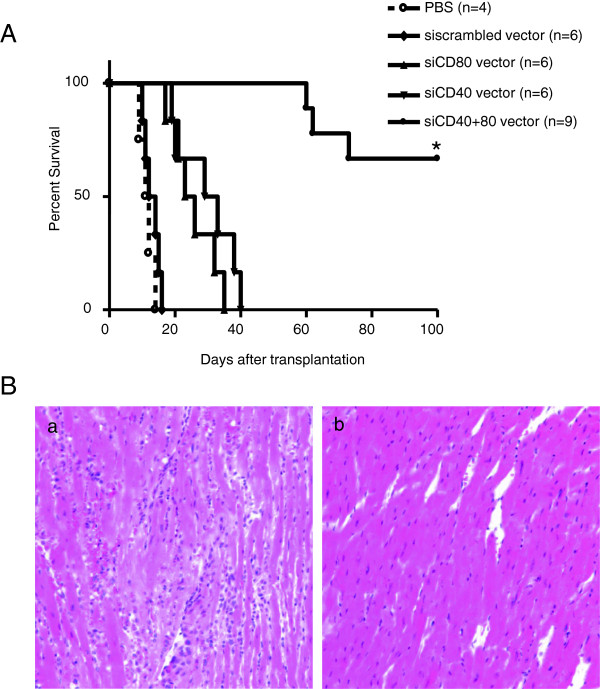
**Prevent graft rejection by using CD40 and CD80 siRNA expression vector. (A)** Allograft survival curve. Recipient BALB/c mice were injected with 50μg of CD40 and CD80 siRNA vectors by hydrodynamic injection through the tail vein at 3 days before transplantation. A low dose (2Gy) of whole body irradiation was administered to the recipient mice before heart transplantation. MHC fully mismatched allogeneic cardiac transplantation was performed from C57BL/6 mice to BALB/c mice. At 7 and 14, 21 days after transplantation, mice were treated with 50 μg CD40 and CD80 siRNA vectors. The groups of mice that were treated with PBS treated or scrambled siRNA vectors were used as controls (*p < 0.05 vs control groups). **(B)** Histopathology of cardiac allograft from recipient mice. Mice were used and treated as described in **(A)**. Cardiac allografts were collected at the time of rejection or 100 days post transplantation. Tissues were sectioned and stained with H&E. Sections from rejected mice **(a)** and CD40 and CD80 siRNA vector treated tolerant mice **(b)** are compared (magnification x200).

Blocking the costimulation pathway by monoclonal antibody can improve allograft survival in rodents and non-human primates [33]. Since our *in vitro* results show that siRNA targeting of CD40 and CD80 reduces costimulatory molecules expression and prevents DC maturation (Figure 1C, D), leading to an inhibition of allogeneic T cell proliferation (Figure 1E), we hypothesized that the blockade of the costimulatory signaling pathway using siRNA expression vectors would prevent graft rejection. To determine this, we treated BALB/C recipients with CD40 and CD80 siRNA vectors before and after fully MHC-mismatched transplantation of C57BL/6 hearts. A low dose (2Gy) of whole body irradiation was administered to the recipient mice before heart transplantation. As expected, untreated recipients or scrambled siRNA vector treated recipients had rapid graft rejection, allografts only survived 12–16 days. Treatment with single CD40 or CD80 siRNAs significantly prolonged cardiac allograft survival (25.7 ± 2.7 days CD80, 30.0 ± 3.6 days CD40) (Figure 3A). Furthermore, combined use of CD40 and CD80 siRNA vectors had synergistic effects of further increasing allograft survival (88.3 ± 5.9 days), while 66.7% of recipients achieved tolerance to allgeneic cardiac grafts (Figure 3A).

At end of point of experiment, cardiac graft tissues were harvested, the pathological changes in the allografts were examined (Figure 3B). The rejected hearts (Figure 3B-a), demonstrated severe cellular and humoral rejection, indicated by lymphocyte infiltration, hemorrhage, infarction and thrombosis. Opposed to the rejected mice, the grafts from the tolerant mice treated with CD40 and CD80 siRNAs showed minimal pathological changes. There was no cellular infiltration, infarction nor thrombosis (Figure 3B-b). These results show that CD40 and CD80 silencing can prevent induce cardiac allograft rejection and induce allograft tolerance.

### Knockdown of costimulatory molecules increases Treg number and function

**Figure 4:**
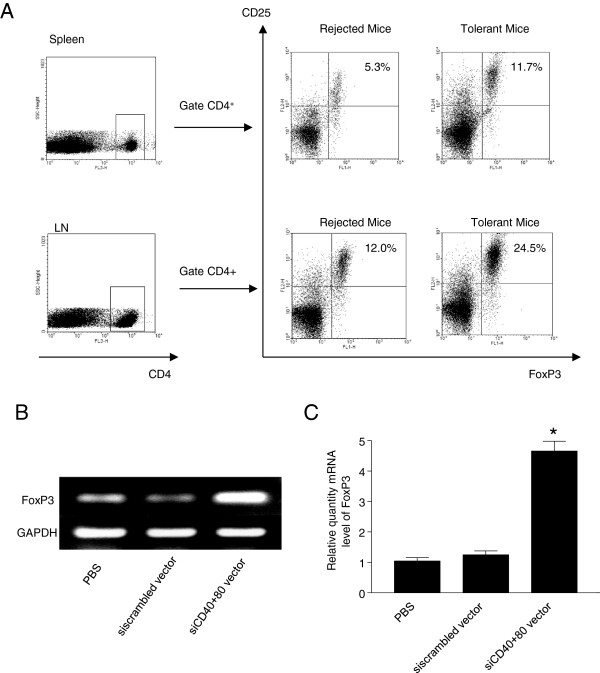
**Treg cells in cardiac allograft recipients. (A)** Flow cytometric analysis of Treg cells in BALB/C recipients with C57BL/6 grafts. T cells were isolated from spleens and lymph nodes of recipient at the time of allograft rejection or 100 days post transplantation. T cells were stained with antibodies against FoxP3, CD25 and CD4. Flow cytometry was performed to determine the percentages of Treg cells by first gating on CD4^+^ cells and then subsequently analyzing the percentages of CD25^+^ and FoxP3^+^ cells in the spleen and lymph nodes of recipients **(A)**. **(B & C)** FoxP3 expression in splenocytes of recipients. T cells were isolated from spleens of recipient mice at the time of allograft rejection or 100 days post transplantation. Total RNA was extracted and transcripts of FoxP3 were determined using RT-PCR **(B)** and quantitative RT-PCR **(C)**. (*p < 0.01 vs control groups).

In order to clarify whether Tregs are involved in maintaining immune tolerance, we identified Tregs in the tolerant and rejecting recipients. There were significantly more Tregs in mice that were treated with CD40 and CD80 siRNA compared to rejected mice (Figure 4A). The numbers of CD4 + CD25 + FoxP3+ Tregs, were significantly increased in the spleens and lymph nodes (LNs) of tolerant mice treated with the combination of CD40 and CD80 siRNA (Figure 4A). The PCR and RT-PCR results demonstrated that FoxP3 expression was significantly increased in the spleen of tolerant treated mice compared to scrambled siRNA treated mice (Figure 4B, C). Collectively, knock down of both CD40 and CD80 costimulatory molecules by siRNA can generate tolerogenic DCs and Treg cells that induce alloimmune tolerance in heart transplantation [34].

**Figure 5:**
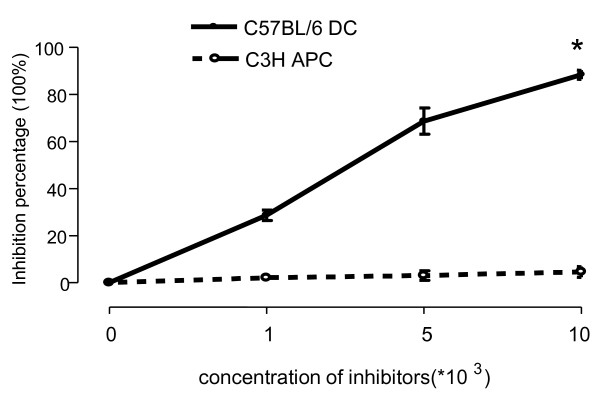
**Inhibitory function of Treg cells from tolerant recipients in MLR assays.** Splenic T cells (2 × 10^5^/well) from naïve BALB/C mice were used as responder cells. Cultured bone marrow DCs (1 × 10^5^) from naïve C57BL/6 and C3H (third party) mice were used as stimulators. CD4^+^CD25^+^ cells from spleens of tolerant recipient mice were added to the cultures, the ratios of Treg cells compared with stimulators were 1:100, 1:20, 1:10. [^3^H]-thymidine incorporation was measured as described in Figure 1E. Inhibition rate was compare with a control where no inhibitor was added in the MLR. Data are presented as the mean ± SEM (*p < 0.01 C57BL/6 DCs vs C3H DCs, n = 6).

In order to determine the specificity of Treg function, we performed inhibitory MLR in the presence of Treg cells. CD4^+^CD25^+^ Treg cells isolated from tolerant recipients (BalB/c) can inhibit donor (C57BL/6) DCs stimulating proliferation in naïve allogenic T cells (BalB/C) in a dose depend manner. However, the CD4^+^CD25^+^ Treg cells from tolerant recipients showed no inhibition of T cell proliferation stimulated by DCs culture from third party (C3H) mice. The data demonstrate that Treg inhibition of MLR occurred in a donor antigen-specific manner (Figure 5).

### DCs in tolerant recipient suppress T cell responses and induce Treg generation

**Figure 6:**
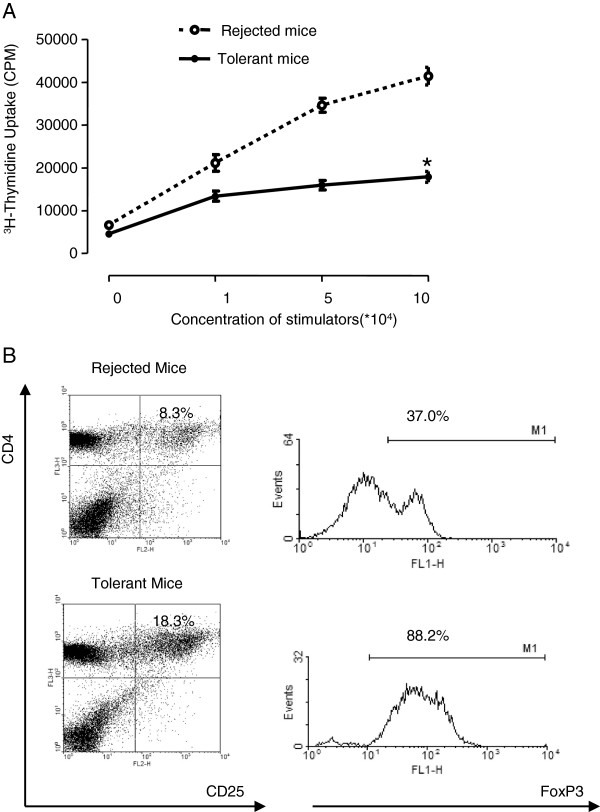
**DCs capacity in cardiac allograft recipients. (A)** DCs from tolerant recipients attenuate the alloimmune stimulatory capacity. Mice were treated and transplanted with allografts as described in Figure 3. Splenic DCs were isolated from BALB/c recipients at the time of rejection or 100 days post transplantation. DCs were cocultured with allogeneic T cells from naïve C57BL/6 mice at varying ratios. After 48h, [^3^H]-thymidine was added to the coculture for another 18h, and its incorporation was measured as an indicator of T cell proliferation. Data are presented as the mean ± SEM (*p < 0.01 vs rejected groups, n = 6). **(B)** Splenic DCs from recipients were cocultured with allogeneic T cells as described in 6**A**. Seven days after the coculture, T cells were stained with antibodies against FoxP3, CD25 and CD4. Flow cytometry was performed to determine the expression of FoxP3 by gating on CD4^+^CD25^+^ cells and then subsequently analyzing the expression of FoxP3.

In the context of transplantation, DCs play a pivotal role in determining the balance between immunity and tolerance [5]. DCs have the capacity to present allograft antigen to recipient T cells to induce graft rejection or acceptance depending on state of DCs. After exposure to the antigen, DCs capture the antigen and express the high level of costimulatory molecules and stimulate T cell responses. Suppression of costimulatory molecules can generate tolerogenic DCs induce more Treg generation and tolerance [35]. It is important to know the state and function of DCs in the recipients. To test this, we first determined the allostimulatory capacity of splenic DCs in tolerance and rejecting recipients. The DCs from recipients with rejected allografts displayed a vigorous stimulation of allogeneic T cell proliferation. In contrast, DCs isolated from the long-term allograft survival recipients treated with CD40 and CD80 siRNA had significantly suppressed T cell responses in a MLR (Figure 6A). To further confirm the feedback loop between tolerogenic DCs and Tregs [34], we isolated splenic CD11C^+^ cells from tolerant or rejected recipient and cultured them with naïve allogeneic T cells for 7 days. The results showed that splenic DCs isolated from tolerant recipient can generate more FoxP3^+^ Treg cells than DCs isolated from rejected mice (Figure 6B). These data demonstrated that knock down of the costimulatory molecules in DCs may generate tolerogenic DCs and induce Treg cell differentiation leading to immune tolerance.

## Discussion

Gene silencing offers the possibility of downregulating genes of interest in a specific and potent manner. Previous studies by our group have demonstrated that immature DCs, or DCs whose costimulatory molecules are silenced, are capable of promoting donor-specific tolerance, in part through induction of Treg cells [27]. In the current study, we sought to utilize a clinically translatable approach, by targeting costimulatory molecules in the recipient through systemic administration of siRNA expressing vectors using hydrodynamic administration. We utilized DCs *in vitro* as a model to assess whether the siRNA that we generated was sufficient for downregulating expression of CD40 and CD80. These molecules were chosen based on previous studies showing importance of these costimulators in blocking transplant rejection [36,37]. We observed that siRNA treatment resulted in specific downregulation of CD40 and CD80 molecules, without non-specific activation of the DC. Furthermore, *in vitro* modulation of DC function was observed such that silenced stimulator DCs were hypoimmunogenic as compared to scrambled siRNA treated DCs in MLR. An additive suppressive effect was seen in MLR when CD80 and CD40 siRNA were simultaneously to treat stimulator DCs.

Gene silencing of DCs was also observed *in vivo* subsequent to hydrodynamic administration of siRNA expression vector. Splenic DCs isolated from siRNA treated mice possessed specific suppression of CD40 or CD80 expression, subsequent to treatment with their respective siRNA sequences. It may be possible that hydrodynamic administration of siRNA vectors resulted in downregulation of costimulatory molecules on other cells as well, as it has been found that endothelial cells express both CD40 and CD80 and these molecules may be involved in allograft rejection [38]. Indeed, previous studies have demonstrated that hydrodynamic administration of siRNA results in endothelial cell transfection [39]. We plan to assess whether silencing in other cells besides DC occurs.

The demonstration of extended allograft survival by recipient treatment with siRNA vector suggests the possibility of developing clinically-relevant protocols for induction of transplantation tolerance. While clinical implementation of hydrodynamic administration is not practical, a more feasible means of recipient modification may be through administration of DC targeted immunoliposomes, which was previously demonstrated by our group [40].

The demonstration of prolonged allograft survival by targeting of recipient costimulatory molecules suggests the possibility of inhibiting indirect antigen presentation. In the process of direct antigen presentation, donor MHC alloantigens are recognized by alloreactive T cells which are found in relatively high frequencies between, 1:100 and 1:10,000 T cells in humans [41]. In contrast, the process of indirect antigen presentation involves recipient antigen presenting cell uptake of the donor antigen, processing of the antigen, and presentation of peptides in the context of self MHC. The frequency of alloreactive T cells with specificity for antigens presented through the indirect pathway is significantly less than for direct antigen presentation, which occurs with a frequency of T cells between 1:100,000–1:1,000,000 T cells [42]. Accordingly, the large number of existing T cells in the direct antigen presentation pathway leads to relatively rapid allograft rejection. In our previous study, ex vivo perfusion of siRNA solution into heart graft effectively attenuated ischemia/reperfusion injury and protected cardiac function [43]. It has not yet been reported the feasibility of perfusing allografts ex vivo using siRNA for prevent immune rejection. Indeed, perfusion of the allograft *ex vivo* might lead to knocking down costimulatory molecules in donor-derived DC thus blocking the direct pathway of rejection. However, this strategy is not able to block the recipient's DC-medicated indirect pathway which induces chronic rejection. Acute rejection in this scenario is effectively controlled by clinical immune suppressants, however, chronic rejection appears to be resistant to current immune suppressants and is the major cause of graft failure today [44]. Given that the mechanism of extended graft prolongation in our study was obtained via the manipulation of recipient antigen presenting cells, we propose that this approach of manipulating the recipient may be more effective at preventing chronic graft rejection in the future. This is supported by the histological observations of reduced signs of chronic rejection such as hemorrhage, infarction and thrombosis.

Mechanistically, prolongation of allograft survival by the CD40 and CD80 combination may be associated with development of a “tolerogenic feedback loop” between Treg cells and DC [34]. In this scenario, hydrodynamic delivery of siRNA-expression vector by systemic administration may suppress the costimulatory molecules on DCs from donor grafts or DCs in recipients. For example, we have identified tolerogenic DCs in tolerant recipients that demonstrated attenuated the alloimmune stimulatory capacity (Figure 6A). These tolerogenic DCs would result in generation of Treg cells, which then would further induce an immature state in the DCs. Such tolerogenic loops have been previously demonstrated through induction of immature DC by blockade of IkB together with Treg stimulation by antiCD45 antibodies [34]. Indeed the possibility of amplifying such tolerogenic loops by administration of agents that increase the number of Treg cells, which has previously been clinically applied using non-Fc binding antiCD3 [45], may be assessed in future experiments to augment the tolerogenic process.

In conclusion, the current paper provides proof of concept for the utilization of siRNA in modifying recipient responses to allogeneic transplantation. The possibility of inhibiting chronic rejection through targeting the indirect pathway of antigen presentation suggests a possibility to overcome limitations of current immune suppressants.

## Abbreviations

DC: Dendritic cell; siRNA: Small interfering RNA; FoxP3: Forkhead box P3; APC: Antigen presenting cell; MHC II: Major histocompatibility complex class II; CTLA4: Cytotoxic T-Lymphocyte Antigen 4; MLR: Mixed leukocyte reaction; Treg cell: T regulatory cell; H & E: Hematoxylin and eosin.

## Competing interests

The authors of this manuscript have no conflicts of interest to disclose.

## Authors’ contributions

Xu Z, YL, GZ: performed experiments and wrote the manuscript. DL: performed heart transplantation surgery. AJ, KS and RC: helped with sample collections. DK and NJ: edited the manuscript. JS, XZ, Xi Z, ZZ, DQ, and WM: study design and edited the draft manuscript. All authors read and approved the final manuscript.
